# Advances of Commercial and Biological Materials for Electron Transport Layers in Biological Applications

**DOI:** 10.3389/fbioe.2022.900269

**Published:** 2022-05-31

**Authors:** Zhifu Yin, Biao Lu, Yanbo Chen, Caixia Guo

**Affiliations:** ^1^ School of Mechanical and Aerospace Engineering, Jilin University, Changchun, China; ^2^ The State Key Laboratory of Alternate Electrical Power System with Renewable Energy Sources, North China Electric Power University, Beijing, China; ^3^ Presidents’ Office of China-Japan Union Hospital of Jilin University, Jilin University, Changchun, China

**Keywords:** electron transport layer, perovskite solar cells, commercial materials, biological materials, biological application

## Abstract

Electron transport layer (ETL), one of the important layers for high-performing perovskite solar cells (PSCs), also has great potential in bioengineering applications. It could be used for biological sensors, biological imaging, and biomedical treatments with high resolution or efficiency. Seldom research focused on the development of biological material for ETL and their application in biological uses. This review will introduce commercial and biological materials used in ETL to help readers understand the working mechanism of ETL. And the ways to prepare ETL at low temperatures will also be introduced to improve the performance of ETL. Then this review summarizes the latest research on material doping, material modification, and bilayer ETL structures to improve the electronic transmission capacity of ETLs. Finally, the application of ETLs in bioengineering will be also shown to demonstrate that ETLs and their used material have a high potential for biological applications.

## Introduction

Perovskite solar cells, which appeared in 2009, have been considered the most promising solar cell in the future. As a new generation of solar cells, PSCs have the characteristics of simple process, clear structure, and low cost, and have therefore become a research hotspot (Mao et al., 2017). PSCs cannot meet the high-power output, but their dexterity makes them have a good application prospect in wearable devices. The typical structure of PSCs consists of a transparent conductive electrode, electron transport layer, perovskite absorption layer, hole transport layer (HTL), and metal electrode.

**FIGURE 1:**
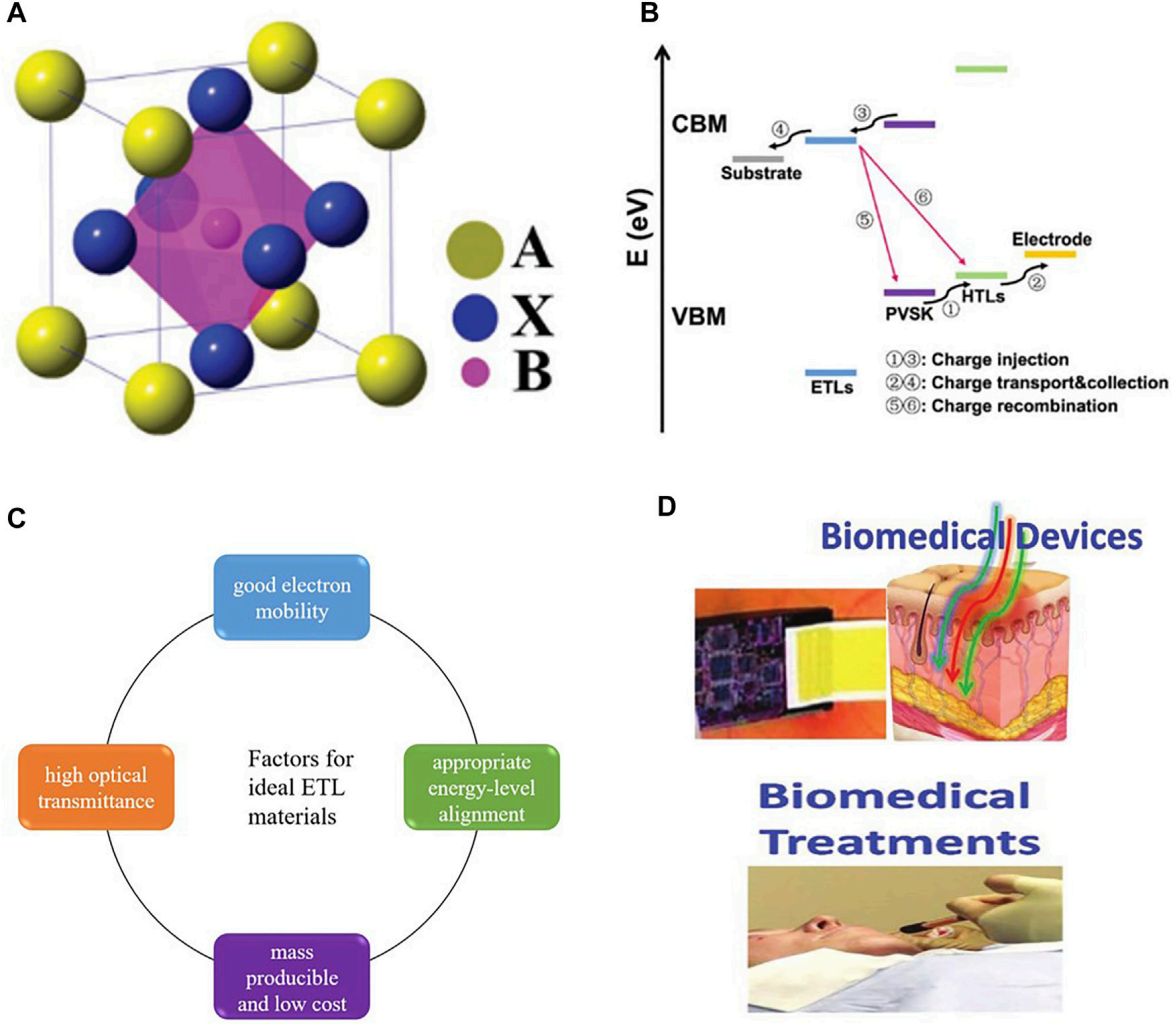
**(A)** Perovskite crystal structures with the general formula of ABX_3_. **(B)** Schematic diagram of the charge transfer process in ETL. **(C)** Factors for ideal ETL materials. **(D)** Applications of PSCs in biomedical devices and biomedical treatment.

Perovskite materials are the most important components of PSCs as light-absorbing materials. The structure of perovskite material is usually composed of three elements, and its structural formula is ABX_3_ (Figure 1A). Cation A, which is most commonly organic-cation, belongs to CH_3_NH_3_^+^, and CH_3_CH_2_NH_3_^+^. Cation B usually refers to divalent metal cations, like Pb_2_^+^ and Sn_2_^+^. Halogen anion X includes three halogen anions: CI^−^, Br^−^, and I^−^ (Hao et al., 2014; Pellet et al., 2014; Liu et al., 2015). Of course, Perovskite materials composed of different ions show great differences in physical and chemical properties. Perovskite materials have many advantages as absorbing materials for solar cells, including high absorption coefficient, adjustable band gap, long carrier diffusion length, and small exciton binding energy (Sun et al., 2014).

In PSCs, the main function of ETL is to extract and transport the photogenerated electrons (Figure 1B) and prevent the recombination of holes and electrons at the interface (Zhou et al., 2020). More importantly, ETL directly determines the performance and stability of the device. Suitable electronic layer materials are supposed to meet the following criteria: 1) good electron mobility, which can ensure the efficient transmission of electrons from the perovskite layer to the electrode, 2) high optical transmittance to reduce the energy loss, 3) appropriate energy-level alignment for electron transfer and hole blocking, 4) mass-producible and low cost (Pathak et al., 2014; Chueh et al., 2015; Chen et al., 2019) (Figure 1C).

Up to now, metal oxides are the most common materials used in perovskite solar cell ETL, due to their high electron mobility, good energy level structure, and other excellent properties. And the semiconducting optoelectronic properties and structural tunability of organic compounds also make them widely used in the electron transport layer of PSCs. In addition, the biologically derived materials in ETL have been applied in recent years. Biological materials can assist perovskite in photoelectric conversion and improve photovoltaic performance. The application of biomaterials in ETL is promising research. And the practicality of biomaterials in photovoltaic devices makes it possible to develop novel biological perovskite cells with high performance and high stability. Besides, the application of perovskite materials in bioengineering is an important factor in the development of new bio-PSCs. The biocompatibility of perovskite materials makes PSCs have applications in biological detection, biological imaging, and biomedical treatment (Figure 1D).

In this review, we will introduce the structure and commercial materials of electron transport layers in PSCs to help readers understand the recent development of ETL. Then, we will introduce the connection between the ETL of PSCs and bioengineering from two aspects, one is the bio-derived material applied in ETL, and the other is the application of ETL in bioengineering.

## The Structures for PSCs

According to the different arrangement orders of ETL and HTL, PSCs architecture can be divided into two types: n-i-p and p-i-n structure (Basumatary and Agarwal, 2022). Due to the difference of ETL structure, n-i-p type PSCs structure contains the planar structure and mesoporous structure. The mesoporous ETL structure includes a mesoporous layer which is mostly composed of TiO_2_ sintered at high temperature. The mesoporous layer can not only act as a scaffold, but also increase the contact between the ETL and the perovskite layer and enhance the charge transport efficiency. In the preparation of PSCs, the material and structure of the electron transport layer have a great impact on the performance and stability of the device (Li et al., 2022). Therefore, a great deal of work has been done in this direction.

**FIGURE 2:**
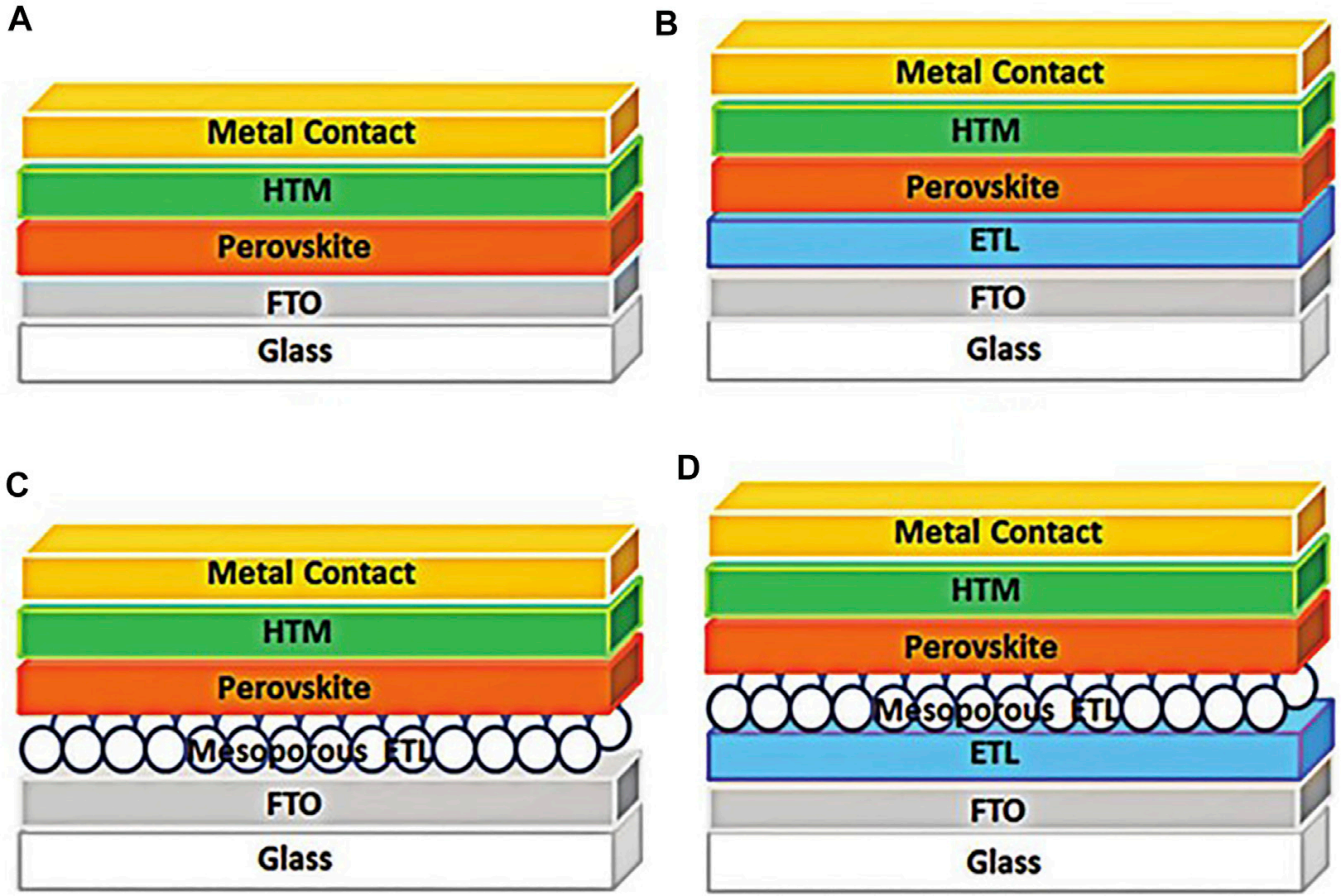
The typical structures of the perovskite solar cells, **(A)** the planar PSCs without ETL, **(B)** the planar PSCs with compact ETL, **(C)** the mesoporous PSCs without compact ETL, **(D)** the mesoporous PSCs with compact ETL.

The current research about PSCs structure mainly includes the ETL free structure, the planar heterojunction of PSCs, the mesoporous configuration of the PSCs without compact ETL, and the combination of compact and mesoporous ETL (Mohamad Noh et al., 2018). Figure 2 shows a schematic diagram of these structures. And most of the PSCs device structures which achieved high efficiency are a combination of compact and mesoporous ETL.

In the n-i-p type PSCs structure, the electron transport layer plays an important role in PSCs. Zhang et al. (2015) reported an ETL-free structure, which behaved a 2.7% reduction in photoelectric conversion efficiency (PCE) compared to devices with an ETL structure, and the ETL-free PSCs structure cannot output stable power. Although the PCE of ETL-free devices can reach 16.1% (Ke et al., 2015b), problems such as device stability cannot be well solved in such structures.

### Planar Structure

**FIGURE 3:**
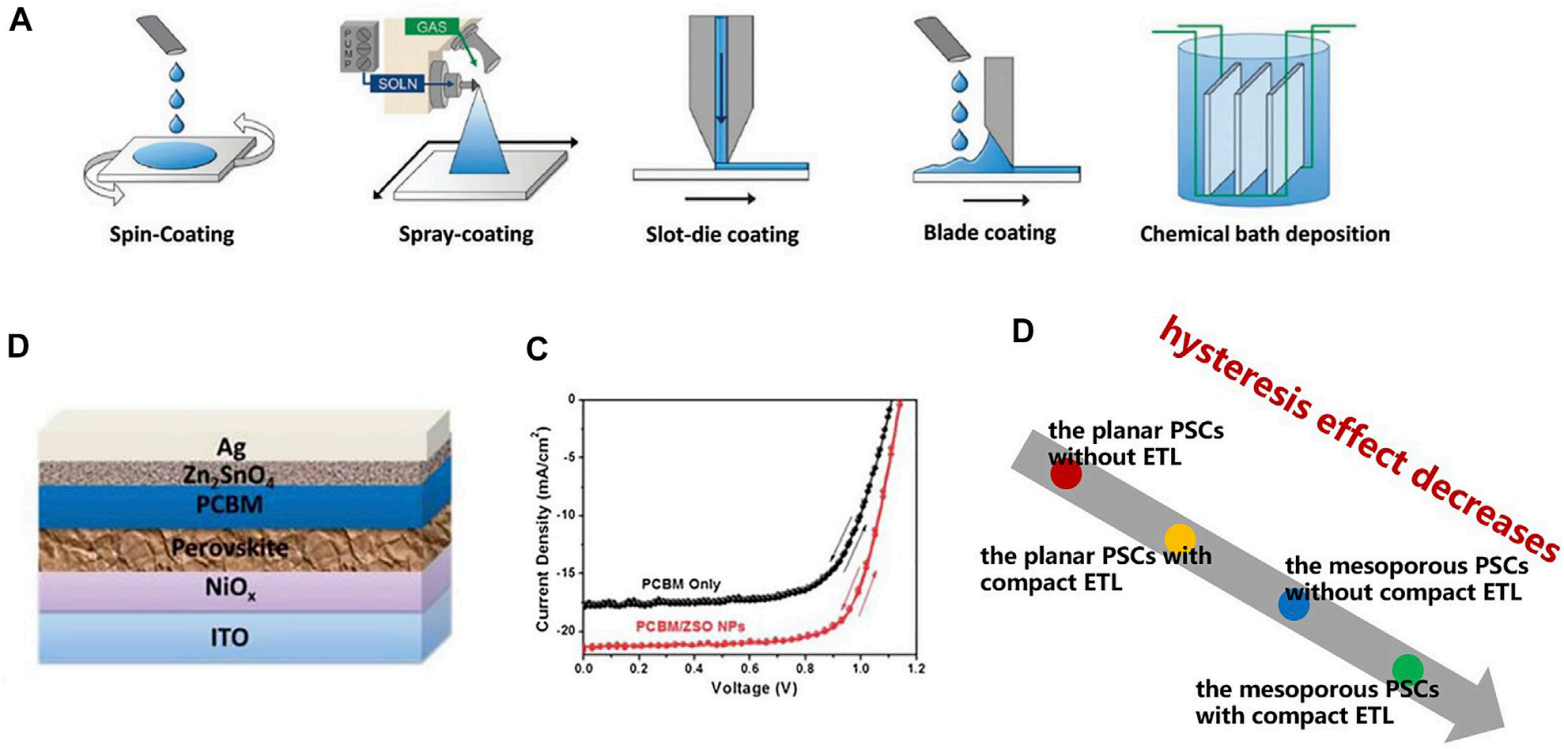
**(A)** Schematic diagram of spin-coating, spray-coating, slot-die coating, blade coating, and chemical bath deposition. **(B)** Schematic diagram of Zn_2_SnO_4_/PCBM double-layer ETL PSCs and its **(C)** J-V curve. **(D)** The effect of ETL structure on the hysteresis of PSCs.

Planar compact ETL structures, commonly used in planar heterojunction PSCs, are mostly deposited on FTO substrates. Compact ETLs are generally fabricated by high temperature or low-temperature deposition processes, but if the substrate is ITO glass, only low-temperature deposition can be used because the resistivity of ITO at high temperatures can be severely damaged. This deposition also includes many experimental methods, including spin-coating, spray-coating, slot-die coating, blade coating, and chemical bath deposition (Altinkaya et al., 2021). Figure 3A shows a schematic diagram of these preparation processes. Meanwhile, ETL prepared by different methods exhibits different morphologies, which will affect the performance of the device. Liu et al. (2013) prepared the first planar PSCs using compact TiO_2_ as ETL with an efficiency of 15.4%. The most likely problem with planar heterostructures is the charge transfer efficiency, which will greatly affect the efficiency of the device.

To improve the performance of PSCs, ETL bilayer is a feasible structure, where one layer has high conductivity and the other layer tends to have better energy levels (Zheng et al., 2019; Lachore et al., 2021). Kogo et al. (2016) prepared a SnO_2_/TiO_2_ bilayer structure. The device showed excellent toughness in several bending cycle tests, and the PCE reached 13.4%. Moreover, the ETL structure of organic/inorganic materials is also applied to planar PSCs, which can inhibit the hysteresis of devices. Liu et al. (2016) added Zn_2_SnO_4_ nanoparticles between the electrode and organic ETL, which enhanced charge transport and passivated perovskite surface defects, resulting in a 5% increase in device efficiency (Figures 3B,C). However, in most studies, the PCE of the planar PSCs structure tends to be lower than that of the mesoporous structure (Cui et al., 2022), which makes the research on PSCs focus on the mesoporous structure.

### Mesoporous Structure

The mesoporous ETL structure includes some gaps, which can allow the electron transport material to have adequate contact with the perovskite layer and enable faster charge transport (Li et al., 2015). At the same time, the mesoporous structure in the form of nanostructures enables better stability of PSCs devices (Mohamad Noh et al., 2018). The mesoporous structure also promotes the growth of perovskite crystals, enabling the formation of thicker absorber layers, which will significantly enhance the absorption of photons. Perovskite solar cells have a multi-layer structure, and the properties and thicknesses of each functional layer are different. Due to the differences in the thickness and properties of each functional layer in PSCs, the coordination of each layer in the preparation process is a problem that needs to be solved. Interface engineering is an approach recognized by scientists. In PSCs containing mesoporous layers, other compact barrier layers (hole blocking layer, HBL) are usually placed under the mesoporous structure to prevent direct contact between the perovskite material and the substrate, leading to electron and hole complex. In PSCs, HBL plays an important role in maintaining the performance of the device. Zhang et al. (2015) reported that the photoelectric conversion efficiency of the mesoporous structure device containing HBL was 10% higher than that without HBL, and it was more stable. This is because HBL can inhibit the transport of photogenerated holes to the PSCs substrate, ensure the transport of photogenerated electrons to external circuits, and improve the electron transport efficiency. Compared with the planar structure, the mesoporous ETL provides space for the growth of perovskite precursors and prolongs the electron diffusion length, which enables PSC devices to achieve high efficiency while maintaining good stability (Lin et al., 2022).

When the irradiated light comes from nothing, it takes a period for PSCs to reach full power output. Similarly, when the irradiated light disappears, there are still electrons stranded inside the PSCs. This phenomenon is called the hysteresis effect, which will seriously affect the efficiency of light energy conversion. The hysteresis of PSCs can be used as a measure of device performance. Some studies have shown that the mesoporous structure can significantly reduce the hysteresis effect compared with the planar structure (Islam et al., 2015). Figure 3D shows the effects of planar structure, and mesoporous structure on the hysteresis of PSCs. It can be seen that the mesoporous PSCs with compact ETL have minimal hysteresis.

## Commercial Materials

Up to now, the commercial materials used in the electron transport layer of PSCs are mainly divided into metal oxides and organic molecules. Both materials have their own advantages and disadvantages. How to give full play to the advantages of the two materials is the research focus of scientists.

**FIGURE 4:**
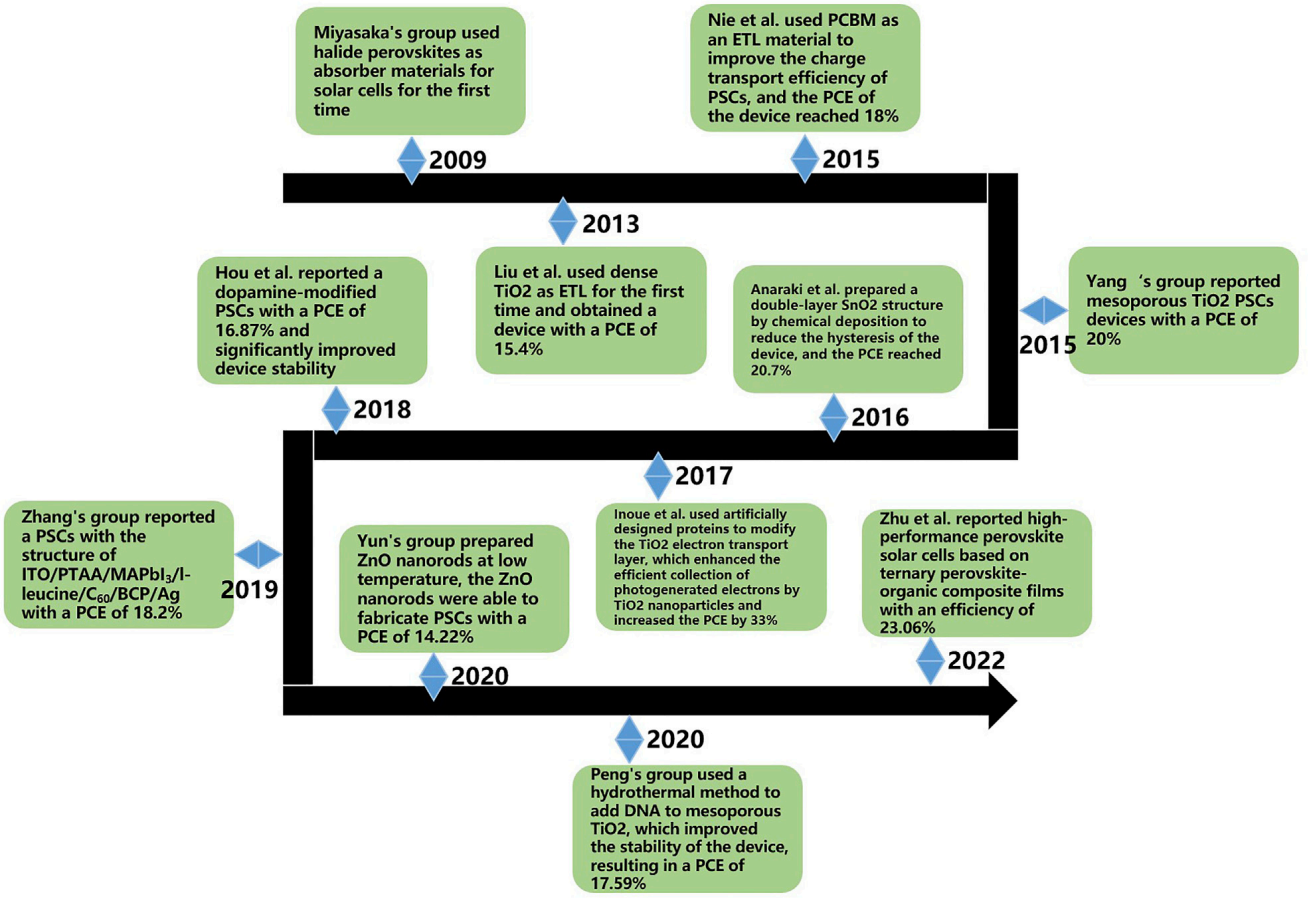
Timeline for the development of ETL materials for perovskite solar cells.

Figure 4 shows a timeline of the past development of materials commonly used for ETL in PSCs. It can be seen that PSCs are developing very rapidly, and device efficiency is increasing at an astonishing rate. Up to now, new materials such as synthetic organic materials and biomaterials have been added to PSC research with satisfactory results. In addition, scientists are working on developing lead-free perovskite devices to reduce environmental hazards. In the future, PSCs may develop towards flexibility and microscale for the commercialization process. PSCs will have broad application prospects in sensors, wearable devices, and biomedicine.

Metal oxide is considered as a promising ETL material because of its high electron mobility and good performance in suppressing the electrical shunt between the transparent electrode and the perovskite layer (Shahiduzzaman et al., 2020). Table 1 summarizes the electronic properties of different metal oxides applied in the electron transport layer of PSCs. And common metal oxide materials used in ETL include titanium dioxide, stannic oxide, zinc oxide, and ternary oxides (Li L. et al., 2018).

**TABLE 1:** Comparison of electrical properties of common ETL materials.

Materials	conduction band minimum[eV]	Energy band gap[eV]	Electron mobility[cm^2^V^−1^s^−1^]	Refractive index	References
TiO_2_	−4.1	3.0–3.2	1	2.4–2.5	Atabaev, (2016)
SnO_2_	−4.22	3.6–4.0	250	2.0	Xiong et al. (2018)
ZnO	−4.17	3.3	200	2.2	Mahmood et al. (2017)
BaSnO_3_	−3.91	3.1	150	2.07	Galazka et al. (2017)
Zn_2_SnO_4_	−4.1	3.8	10–30	2.0	Elseman et al. (2019)

In general, we take photoelectric conversion efficiency as an indicator to evaluate the performance of PSCs. PCE is determined by short-circuit current density (J_SC_), open-circuit voltage (V_OC_), and fill factor (FF). J_SC_, V_OC_, FF, and PCE together constitute the photovoltaic parameters of PSCs. Table 2 summarizes these key photovoltaic parameters of the PSCs prepared by common ETL materials (including dopant materials).

**TABLE 2:** Perovskite solar cells with different structures corresponding to performance parameters of devices.

structure	V_OC_[V]	J_SC_[mA cm^−2^]	FF[%]	PCE[%]	References
FTO/TiO_2_/CH_3_NH_3_PbI_3_/spiro-OMeTAD/Ag	1.65	18.35	67.6	13.15	Xie et al. (2019)
FTO/SnO_2_/CH_3_NH_3_PbI_3_/spiro-OMeTAD/Au	1.15	21.74	80.9	20.23	Zuo et al. (2017)
ITO/ZnO/CH_3_NH_3_PbI_3_/spiro-OMeTAD/Ag	0.99	20.08	56.0	11.13	Son et al. (2014)
FTO/BaSnO_3_(La)/CH_3_NH_3_PbI_3_/PTAA/Au	1.12	23.4	81	21.3	Xia et al. (2015)
ITO/PEDOT:PSS/CH_3_NH_3_PbI_3_/PCBM/Al	0.87	20.7	78	14.1	Seo et al. (2014)
FTO/SnO_2_/Ti O _2_/CH_3_NH_3_PbI_3_/spiro-OMeTAD/Ag	1.10	22.52	76.1	18.85	Xie et al. (2019)
FTO/TiO_2_(Mg)/CH_3_NH_3_PbI_3_/Spiro-OMeTAD/Au	0.80	10.4	50	4.7	Manseki et al. (2014)
ITO/ZnO(In)/CH_3_NH_3_PbI_3_/Spiro-OMeTAD/Ag	1.00	23.00	70	16.10	Mahmood et al. (2018)

### Titanium Dioxide

Titanium dioxide (TiO_2_) is an ETL material in the most reported efficient PSCs for its proper forbidden bandwidth, good optical and chemical stability, nontoxicity, corrosion resistance, and simple manufacturing process (Que et al., 2016). Both mesoscopic and dense TiO_2_ films perform well in PSCs, and their properties can be improved by various modifications and synthesis methods (Xiong Y. et al., 2016; Ullah et al., 2021).

**FIGURE 5:**
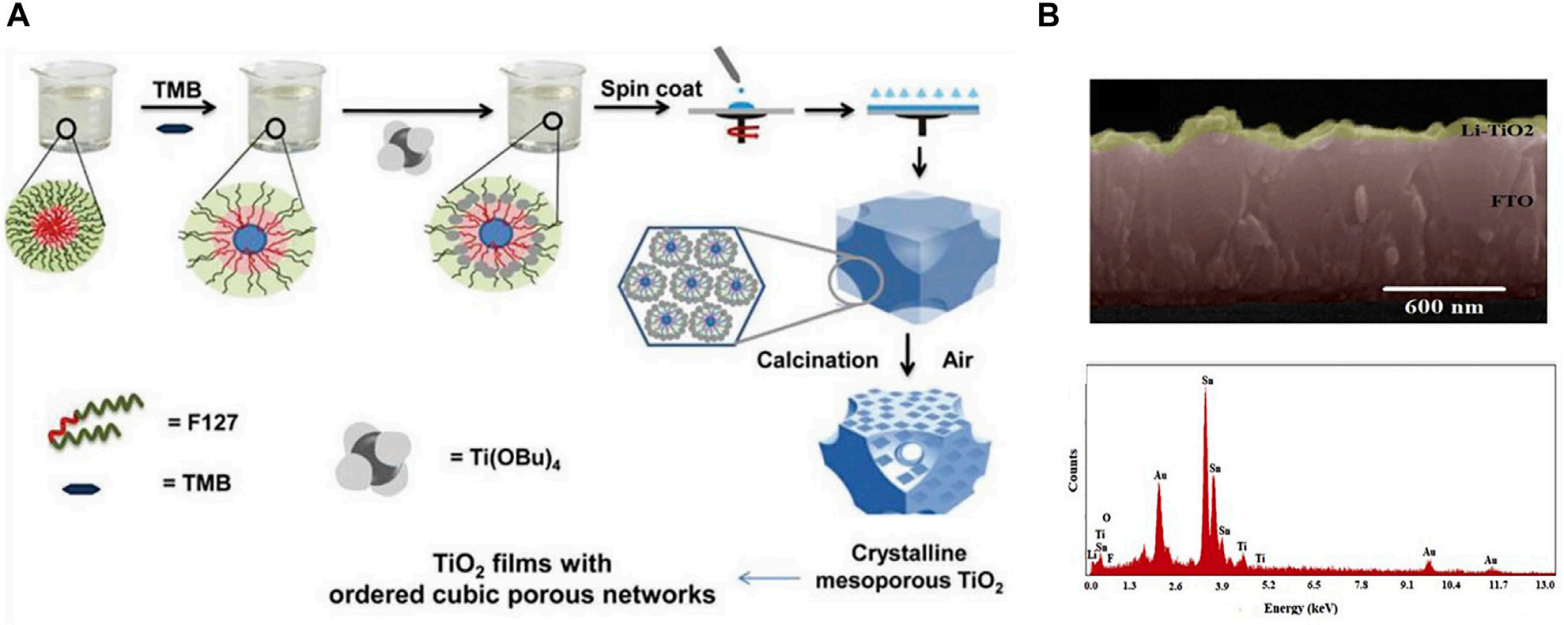
**(A)** Preparation of TiO_2_ films by evaporation induced self-assembly combining the pore-enlarging effects of the swelling agent. **(B)** Cross-section and EDX spectrum of lithium doped TiO2 device.

There are many studies on TiO_2_ mesoporous layer. And the TiO_2_ mesoporous layer is composed of TiO_2_ nanoparticles, which are conductive to the increase of perovskite grain size and enhance light scattering and absorption. Yang reported a cell with 20% efficiency using a mesoscopic TiO_2_ ETL, while another the efficiency of the device without a scaffold layer was only 12% (Yang et al., 2015). Sarkar reported a method for preparing mesoscopic TiO_2_ thin films using pluronic copolymer (F127) and swelling agent, and the best morphological film can be obtained by controlling the ratio of F127/swelling agent, but this method is complicated and expensive (Sarkar et al., 2014). Figure 5A shows the process of preparing TiO_2_ thin films by Sarkar’s group. Subsequently, Yue et al. (2015) used PMMA as a template and prepared a TiO_2_ film with better morphology by a sol-gel method, and the pore size of the film could be changed by changing the amount of PMMA. Ting et al. (2018) found that TiO_2_ nanoparticles with different sizes can affect the efficiency of perovskite solar cells. And they increased the average efficiency of the PSCs by 11.2%, by mixing 0.2 μm and 20 nm TiO_2_ with a ratio of 1:2.

However, the deposition process of the TiO_2_ mesoporous layer is cumbersome, and the high-temperature sintering process above 500°C will increase preparation cost and is not suitable for the preparation of flexible PSCs (Suresh Kumar and Chandra Babu Naidu, 2021). In addition, the electron mobility of TiO_2_ is low (∼1 cm^2^ V^−1^ S^−1^) (Shin et al., 2019), which will lead to the imbalance of electron transmission and affect the performance of the device (Jung and Park, 2015). And the high photocatalytic activity of TiO_2_ makes it very likely to cause damage to the perovskite layer under ultraviolet light conditions. Therefore, many attempts have been made to dope TiO_2_. Through doping, the energy level of the electronic layer can be adjusted, the performance of transport charges can be improved, the conductivity of the film can be improved, and the film can be made more stable, all of which can greatly improve the performance of PSCs (Wang and Liao, 2018; Valadi et al., 2021). Sidhik et al. (2018) mixed the cobalt (Co) ions into the lattice of mesoporous TiO_2_, and got the maximum photoelectric conversion efficiency improvement from 14.92% to 18.16%. Similarly, Teimouri did the effect of lithium (Li) doping on the performance of TiO_2_ ETL, and the results showed that Li-doped TiO_2_ ETL had higher conductivity and faster electron transport, and Li can effectively reduce the hysteresis behavior of PSCs. For different concentrations of Li doping, the device efficiency of 0.3M Li-doped TiO_2_ performed the best (Teimouri et al., 2020) (Figure 5B). Doping has a major effect on the band structure and trap states of TiO2, which will affect the energy conduction and charge transport of the device. Scientists have tried many different dopants, and different synthesis methods, all of which can more or less eliminate TiO2 defects and affect device performance. At the same time, researchers are also studying the TiO_2_ compact layer, which can simplify the preparation process and does not need high-temperature sintering (Wang and Liao, 2018). For the TiO_2_ dense layer, the thickness of the film needs to be optimized to realize electron transport. When the ETL is too thick, the electron transport efficiency will be reduced due to the decrease in electron mobility (Mohammadian-Sarcheshmeh and Mazloum-Ardakani, 2018). In the ETL structure of high-efficiency PSCs, the TiO_2_ dense layer is usually inserted under the mesoporous layer to prevent device shunting.

Up to now, the most efficient PSCs certified are based on TiO_2_. It can be seen that TiO_2_ is an ideal ETL material, which has many advantages over other materials (Dharani et al., 2014). However, the preparation of mesoporous TiO_2_ layers requires a high-temperature sintering process, which not only affects the optical properties of perovskite materials, but also leads to severe hysteresis of the device.

### Stannic Oxide

**FIGURE 6:**
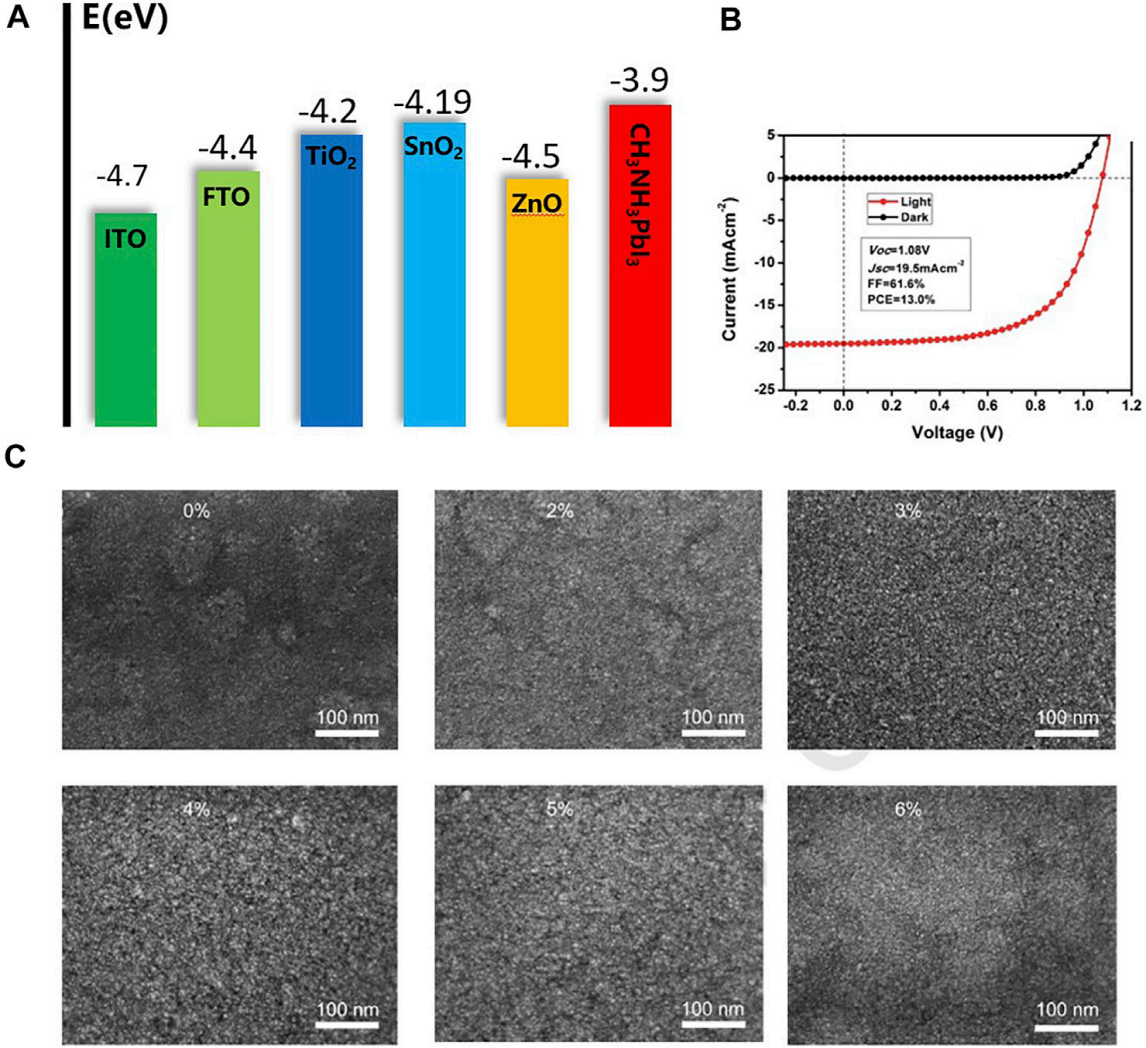
**(A)** Energy level diagram of common ETL materials and other functional layers. **(B)** J-V curves of the highest performing PSCs devices measured in 100 mW cm^−2^ AM 1.5 G illumination and darkness. **(C)** The SEM images of SnO_2_ films (pristine SnO_2_,2% Li: SnO_2_,3% Li: SnO_2_, 4% Li: SnO_2_,5% Li: SnO_2_,6% Li: SnO_2_).

Another metal oxide, stannic oxide (SnO_2_), which has higher electron mobility than TiO_2_, is also a widely used electron transport layer material. Compared with TiO_2_, SnO_2_ has good anti-reflection, excellent chemical stability, and lower photocatalytic activity, which are beneficial to maintain the stability of perovskite devices (Yakovkin and Petrova, 2013). ETL materials should have well-matched energy levels to ensure electron transport and hole blocking. Figure 6A summarizes the energy levels of common ETL materials and other functional layers. In addition, SnO_2_ can be prepared at low temperatures (Guo et al., 2018). The SnO_2_ ETL thin films prepared at low temperature has excellent performance, and the device can achieve the same efficiency as TiO_2_-based PSCs (Shih et al., 2017). Song et al. (2015b) reported a highly stable 13% PCE device through a low temperature treated SnO_2_ ETL (Figure 6B). Ke et al. (2015a) prepared SnO_2_ solution as ETL by a simple method. After UV treatment, spin coating, and annealing, devices with a PCE of 17.21% were obtained, and they maintained good stability under continuous light conditions. In addition, they found that when the FTO substrate was 60 nm, the light transmission effect was great, which may be better for anti-reflection. Anaraki et al. (2016) reported a double-layer SnO_2_ device by chemical bath deposition, the PCE of which achieved 20.7%. And the hysteresis of the device has been well improved. Vijayaraghavan et al. (2020) prepared SnO_2_ quantum dots as ETL materials. SnO_2_ quantum dots have excellent electron transport and hole blocking capabilities, and the prepared PSCs also showed high efficiency. The performance of the ETL can also be improved by doping SnO_2_ with other elements. Huang et al. (2020) added LiCl to the SnO_2_ ETL, obtaining perovskite devices with lower hysteresis and higher stability (Figure 6C). Xiong L. et al. (2016) doped SnO_2_ films with different contents of Mg by high-temperature treatment to adjust the energy level of the electronic layer and broaden the energy generation of SnO_2_. The results showed that the efficiency is nearly doubled compared with the undoped device under the same conditions. After multiple rounds of process optimization by researchers, the PCE of SnO_2_ ETL devices has reached over 23% (Jiang et al., 2019). SnO_2_ is an ideal material for the preparation of PSCs ETL, but a lot of work is still needed to improve film quality, surface defects, etc., which will make charge transport more efficient and improve the efficiency and stability of SnO_2_ devices. Compared with TiO_2_ materials, the biggest advantage of SnO_2_ is its low-temperature processability, which makes it compatible with temperature sensitive substrates and can be used in flexible devices (Park and Zhu, 2022).

### Zinc Oxide

**FIGURE 7:**
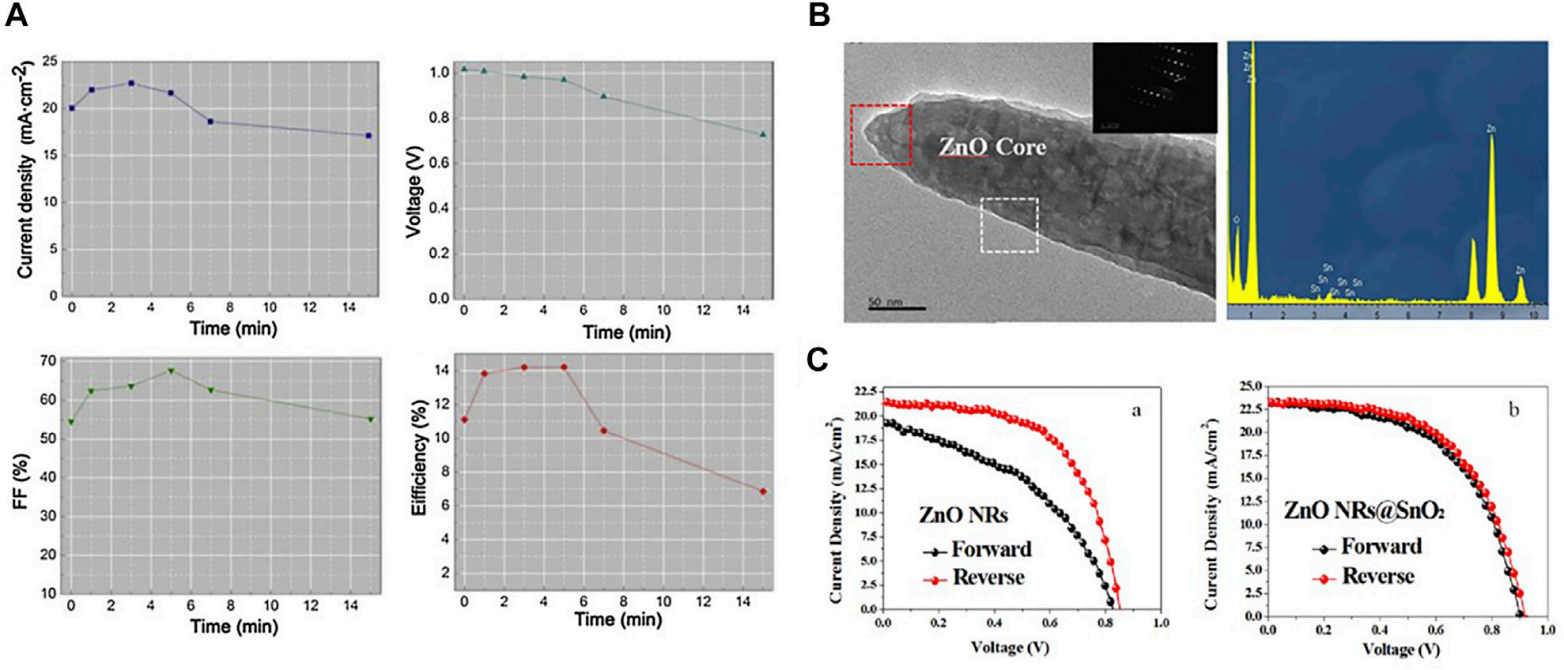
**(A)** Effect of the length of ZnO nanorods on the photovoltaic performance parameters of devices (short-circuit current density, open-circuit voltage, filling factor, photoelectric conversion efficiency). **(B)** The TEM image and simultaneous energy-dispersive spectroscopy (EDS) of SnO_2_ doped ZnO nanorods. **(C)** J-V curves of PSCs with the bare ZnO substrate and the SnO_2_ doped ZnO nanorod substrate which are measured by forward and reverse scan directions.

Zinc oxide (ZnO) has many excellent physical and chemical properties, such as high electron mobility, high transmittance, and good energy level structure (Zhang et al., 2018). Moreover, ZnO can form various nanostructures including nanoparticles and nanorods at low temperatures (Kim T. et al., 2020). ZnO nanoparticles and nanorods as electron transport layers have electron mobilities of 11.13% (Son et al., 2014) and 15.7% (Liu and Kelly, 2013), respectively. But the disadvantage of ZnO is that it is relatively unstable chemically. In addition, the poor chemical compatibility of ZnO with metal halide perovskites makes it difficult to prepare efficient and stable PSCs. Dong et al. (2014) prepared dense ZnO and mesoporous Al_2_O_3_ as ETL by atomic layer deposition, with a PCE of 13% at an effective area of 0.04 cm^2^ and maintained certain stability. Compared with the dense layer, the research on ZnO nanoparticles is more extensive. Song et al. (2015a) prepared ZnO nanoparticle ETL PSCs by spin coating with a PCE of 13.9%, and more importantly, the device efficiency remained stable for 20 days. Yun et al. (2020) grew ZnO nanorods with time-varying lengths on FTO substrates employing a low-temperature water bath. They used them as ETLs, and by adjusting the size of ZnO, the ZnO nanorods were able to fabricate PSCs with a PCE of 14.22%. Figure 7A shows the effect of the length of ZnO nanorods on the optoelectronic parameters of PSCs. Kumar et al. (2013) prepared ZnO nanorods by chemical deposition at low temperature as ETL, and obtained PSCs with great performance. And they have experimentally demonstrated that nanorods have improved performance in all aspects compared to nanoparticles. To solve the problem of perovskite corrosion, Wang P. et al. (2017) doped the SnO_2_ film between the ZnO ETL and the perovskite layers. The results showed that the hysteresis of the device is significantly reduced, and the PCE of the device reached 12.17% (Figures 7B,C). Son et al. (2015) fabricated a layer of TiO_2_ on the surface of ZnO nanorods by solution deposition. And the performance and stability of the device have been significantly improved under the modification of TiO_2_. ZnO has similar physical properties to TiO_2_ and is suitable for ETL preparation. And ZnO has higher electron mobility, which is beneficial to charge transport. In addition, the advantage of ZnO lies in its ability to prepare ZnO nanomaterials in a simple and low-cost way. These nanomaterials can directly form porous and dense films by a simple solution method, which plays a good role in transporting electrons and blocking holes (Ahn et al., 2022). However, compared with the PSCs based on TiO_2_, the performance of ZnO devices is still inferior, and the disadvantages of ZnO materials such as high defect density and low stability need to be optimized.

### Ternary Oxides

Compared with binary oxides, ternary oxides have better performance in certain aspects. For instance, the optoelectronic properties of ternary oxides can be controllably tuned by changing their molecular structures, and they also have better performance in terms of flexibility. Therefore, materials such as BaSnO_3_(BSO) and Zn_2_SnO_4_ (ZSO) can also be used as ETL materials for PSCs. BSO is a kind of semiconductor perovskite oxide, which is a feasible material as ETL. Zhu et al. (2016) first applied BSO to the mesoporous layer of PSCs and obtained good PCE (12.3%). Li X. et al. (2018) used a hybrid ETL layer of BSO nanoparticles and PCBM to prevent the intrusion of air moisture and oxygen, and applied this to inverted PSCs, which improved the PCE of the device by 16.2%. And after 600 h of storage, it still maintained 90% of the initial PCE. ZSO is regarded by many researchers as an ideal ETL material due to its high electron mobility and wide optical bandgap. Jung et al. (2018) tried to use the ZSO film prepared by solution processing as the ETL of the PSCs, and the efficiency of the device reached 20.02%. Shin reported a new method for the preparation of highly dispersed ZSO nanoparticles at low temperatures and introduced it into the preparation of flexible PSCs. The obtained device exhibits steady-state power conversion efficiency (PCE) of 14.85% (Shin et al., 2015). Zhu et al. (2022) reported high-performance perovskite solar cells based on ternary perovskite-organic composite films with an efficiency of 23.06%. The strategy of utilizing ternary perovskite-organic composite thin films could lead to a facile way to realize high-performance perovskite solar cells.

Ternary oxides have various crystal structures, researchers can tune the optical and electrical properties by changing their chemical compositions, which makes ternary oxides have great potential as ETL materials for PSCs. However, ternary oxides tend to be less stable than other oxides such as TiO_2_ and SnO_2_. At the same time, the optimization of the preparation method of ternary oxides needs to be considered.

### Organic Molecules

In recent years, organic molecules as ETL materials have also attracted extensive attention, including C_60_ (Wojciechowski et al., 2015), PCBM (Liu et al., 2019), CDIN (Lian et al., 2018), and some synthetic organic small molecules. Most of these organic small molecule materials have high electron mobility, and they are easily processed and chemically modified, which can meet the conditions of ETL materials. Liu et al. (2018) demonstrated the excellent ability of C_60_ in electron transport and recombination inhibition by vacuum deposition of C_60_ as the ETL of PSCs. And they optimized the thickness of C_60_ to improve the grain boundaries of the perovskite layer. Jeng et al. (2013) used PCBM and ICBA instead of C_60_ as the ETL materials, and the photoelectric conversion efficiency can reach more than 6% by using a low-temperature preparation process. Nie et al. (2015) used PCBM as the ETL material for PSCs and found that the open-circuit voltage and fill factor of the device were significantly improved, and by optimizing the material of the perovskite layer, the PCE reached 18%. Compared with metal oxides, organic molecules have relatively low efficiency in transporting charges, and the problem can be improved by placing a buffer layer between the electrode and the ETL. Wu et al. (2016) spin-coated PF_6_-IL as a buffer layer onto ETL, which improved the PCE of planar PSCs device to 19%. By changing the thickness of the PCBM and inserting a thin layer of LiF to block holes, Seo et al. (2014) prepared PSCs with a PCE of 14.1% at low temperature. Organic molecules can also improve the electronic layer performance by doping. Xia et al. (2015) incorporated an oleamide amphiphilic surfactant into the PCBM film to make the film coverage more uniform and increase the interfacial contact with the perovskite layer. Snaith et al. reported that the stability of C_60_ ETL could be improved by doping dihydro-1H-benzo-imidazol-2-yl (N-DBI) derivatives, the effect was obvious, and this organic material could be removed by solution method (Wang Z. et al., 2017). Table 3 summarizes the photoelectric parameters of the organic-doped PSCs.

**TABLE 3:** Parameters of PSCs containing organic doped ETL.

Device structure	V_OC_[V]	J_SC_[mA cm^−2^]	FF[%]	PCE[%]	References
ITO/PEDOT:PSS/MAPbI_3-X_Cl_X_/Oleamide:PCBM/Ag	0.98	18.76	69	12.69	Sakai et al. (2017)
FTO/C60(N-DPBI)/MAPbI_3-X_Cl_X_/Spiro-OMeTAD/Au	1.06	23.00	75	18.30	Hu et al. (2017)
ITO/NiO_X_/MAPbI_3_/PCBM(DBU)/Ag	1.02	21.54	76	16.69	Chang et al. (2015)
ITO/PEDOT:PSS/MAPbI_3_/PCBM(CTAB)/Ag	1.01	22.41	76	17.11	Huang et al. (2016)
ITO/PEDOT:PSS/MAPbI_3_/PCBM(P1-PF_6_)/Ag	1.03	17.32	71	12.55	Bin et al. (2016)
ITO/NiMgLiO/MAPbI_3_/PCBM(H3)/BCP/Ag	1.06	21.5	79	18.10	Momblona et al. (2016)
ITO/C_60_(Phlm)/MAPbI_3_/TaTm:F_6_-TCNNQ/Au	1.14	22.08	81	20.30	Guo et al. (2016)

Compared with metal oxides, the molecular structure of organic electron transport materials is easy to change, and the flexibility is better. However, most organic materials have low electrical conductivity. To ensure the electron transport efficiency, it is necessary to reduce the thickness of the organic electron layer, or introduce an intermediate layer between ETL and perovskite, which can be achieved by optimizing the preparation process. In PSCs with organic materials as the electronic layer, the efficiency is generally lower than in metal oxide devices (Xiao et al., 2022).

Up to now, the biggest challenge of ETL is how to achieve large-scale production. Common materials have strict requirements for the preparation process. Therefore, it is very important to develop a simple and low-cost ETL preparation process. In addition, due to the instability of perovskite materials, ETL materials need to contribute to the stability of devices.

## Biological Materials

The high performance of PSCs in photoelectric conversion and the low cost in the fabrication process make them have great potential for commercialization. However, in the preparation of PSCs, there are many opportunities for the optimization of materials and processes. For example, the selection of perovskite materials is limited, and the problems of toxicity and instability of materials are difficult to solve (Pellet et al., 2014). To solve the problems arising in PSCs, scientists have carried out many studies through grain adjustment, interface modification, etc. At this time, the application of some biologically derived materials with polar groups into ETL has aroused the interest of scientists, mainly because they can improve the morphology of the deposited perovskite, increase the grain size, and reduce the surface defects. In addition, these materials can modify the ETL/perovskite heterojunction interface and increase the charge extraction efficiency (Cavinato et al., 2021). Table 4 summarizes the performance of PSCs prepared by several typical biological materials.

**TABLE 4:** Performance of biologically derived PSCs.

compound	V_OC_[V]	J_SC_[mA cm^−2^]	FF[%]	PCE[%]	References
Glycine	0.98	16.28	60	9.48	Shih et al. (2015)
Alanine	0.99	22.40	64	14.22	Shih et al. (2017)
Cysteine	1.11	23.6	70	18.3	He et al. (2018)
Bacteriorhodopsin	1.05	22.61	70.5	17.02	Das et al. (2019)
Norepinephrine	0.97	20.58	72	14.4	Huang et al. (2017)
DNA	1.07	22.90	71.9	17.59	Peng et al. (2020)
Dopamine	1.05	21.72	71.8	16.31	Hou et al. (2018)
Polydopamine	1.02	18.19	65.14	12.05	Huang et al. (2018)

### Amino Acids

Amino acids are organic compounds containing carboxyl, amino and specific side groups. In photovoltaic cells, amino acids can be used as surface passivation additives or dipole surface enhancers and have some applications in the processing of thin films.

**FIGURE 8:**
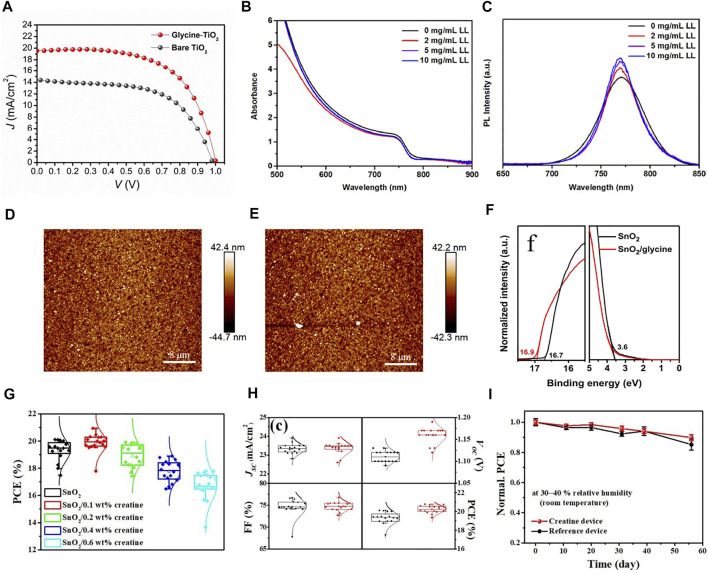
**(A)** J-V curves of perovskite solar cells with bare TiO_2_ and glycine treated TiO_2_. **(B)** UV-Vis and **(C)** PL spectra of perovskite films fabricated using different concentrations of l-leucine. AFM images of SnO_2_ **(D)** and modified SnO_2_ **(E)**. **(F)** UPS spectra of SnO_2_ and SnO_2_/glycine thin films on ITO substrates. **(G)** Histogram of the PCE of the initial optimization of the concentration of creatine precursor. **(H)** Histograms of all parameters for reference device (black) and 0.1 wt% creatine device (red). **(I)** Stability changes of 0.1 wt% creatine device and control group.

Amino acids have been confirmed as additives to improve the performance of PSCs, and it has been reported that surface modification of TiO_2_ with amino acids can significantly improve the stability of PSCs (Shih et al., 2017). The amino group of amino acid can interact with the perovskite layer, and the peptide can act as an intermediate layer to enhance the electronic coupling with the perovskite layer. In addition, amino acids can enable the preferential growth of perovskite crystals and improve the crystallinity of perovskite materials. Shih et al. (2015) used amino acid glycine as the intermediate layer to improve the surface defects in the crystallization process of perovskite, and significantly improved the performance of the PSCs in all aspects. In the case of glycine treatment, the grain size is smaller and the contact area between layers is wider. Figure 8A shows that the performance of TiO_2_ devices treated with glutamate is significantly better than that of untreated devices. Since then, scientists have studied the effect of using different other amino acids (alanine, cystine, leucine, etc.) to modify TiO_2_ ETL. The main research point is the effect of amino acid addition on charge transport and perovskite layer crystallization. Zhang W. et al. (2019) used l-leucine to improve charge transport in PSCs and prevent water/oxygen in the air from entering the device interior, which is due to the formation of a passivation layer by l-leucine. Among the PSCs with the structure of ITO/PTAA/MAPbI_3_/l-leucine/C_60_/BCP/Ag, the highest PCE can reach 18.2%, which is significantly better than the control group. Figures 8B,C shows the UV-Vis and PL spectra of leucine-treated PSCs at different concentrations. He et al. (2018) soaked the ITO substrate covered by Cu-doped NiO electrode in amino acid cysteine for 12 h, and the surface wettability of the treated Cu:NiO_x_ film was improved, which is conducive to the formation of uniform defect free perovskite films. Similarly, Wu et al. (2020) used l-cysteine to modify the TiO_2_/CH_3_NH_3_PbI_3_ interface, which increased the PCE from 11.5% to 14.4%, and the J_SC_ and V_OC_ of the device also increased significantly. Studies have shown that this is due to the interaction between functional groups (–COOH, –NH_2_) in l-cysteine and Ti^4+^ ions in TiO_2_ and free Pb^2+^ and I^−^ in the perovskite layer, thereby improving the transfer efficiency of electrons from the perovskite layer to the ETL. During the preparation of PSCs with SnO_2_ as ETL, low temperature annealing may lead to the formation of lots of oxygen vacancies. To solve the related problems, amino acid modification of SnO_2_ has been reported. Wang et al. (2022) treated SnO_2_ colloids with glycine to prepare dense SnO_2_ ETL films, which reduced the non-radiative recombination at the perovskite/ETL interface and obtained high-performance and high-stability PSCs devices. Han et al. (2021) added alanine to the precursor solution of SnO_2_ films, which reduced the vacant oxygen sites, improved the quality of SnO_2_, and increased charge transport. Furthermore, the amino group of alanine could promote the crystallization of halide perovskite films through hydrogen bonds, reduce carrier recombination, and improve the stability of the device. Figures 8D,E shows the AFM images of SnO_2_ film treated with alanine and the untreated film. It can be seen that the roughness of the modified SnO_2_ film is significantly reduced. Du et al. (2020) reported the modification of SnO_2_ ETL by amino acids and the effect on perovskite crystallization. They added glycine into PSCs as a buffer layer through a simple low-temperature preparation process, releasing the potential of SnO_2_ ETL-based PSCs, significantly improving the quality of perovskite films, with the best efficiency reaching 20.68%. Figure 8F is the UPS spectra of SnO_2_ and SnO_2_/glycine films, it can be seen that the energy level of SnO_2_ changed after glycine treatment. The great advantage of this experiment is the low-cost, low-temperature processing preparation process. Another approach to improve device performance is to incorporate self-assembled monolayers (SAM), which are composed of an anchoring group and a functional group. The SAM as the active layer can control the growth and structure of the perovskite. Kim G.-W. et al. (2020) added 2-[carbamimidoyl (methyl)amino] acetic acid (commonly known as creatine) in SnO_2_ layer. The ammonium and amine groups in creatine can promote the uniform formation of perovskite films and improve film defects. The PCE of the device optimized by creatine layer reached 22.1% and maintained an initial efficiency of 90% after 50 days Figures 8G–I shows that the 0.1 wt% creatine device performed the best. At the same time, the V_OC_ and PCE values of the creatine-added device increased compared to the untreated device, and the stability was also improved. Kouki et al. (2022) synthesized a new molecule, HOOC−(CH_2_)_n_−PP−(CH2)_n_NH_3_^+^Cl^−^, and grafted it onto ZnO layer as SAM, making the crystallinity of perovskite better and the stability of the device stronger.

Up to now, amino acids are mostly used as additives in the structure of PSCs, where they can improve surface passivation and enhance charge transfer efficiency (Hu et al., 2022). Since the optical properties of amino acids do not meet the conditions of ETL materials, they cannot be used as PSCs structural materials alone. Scientists’ research is to use amino acids to treat metal oxides or other PSCs materials to achieve the purpose of improving device performance. In addition, the experimental steps of amino acid surface modification still have room for optimization. For example, we need to consider how to reduce the cost and ensure the low temperature environment in the preparation process.

### Proteins

Protein is an organic macromolecule composed of one or more polypeptides. Proteins play important roles in living organisms, such as catalysts (enzymes), transport function (hemoglobin), information transmission (insulin), and immune function (antibodies). Due to the rapid development of genetic engineering technology, scientists have been able to synthesize artificial proteins with various functions, and this protein-based method can be easily utilized in the fabrication of other nanodevices, such as batteries, supercapacitors, and biosensors, etc.

Inoue et al. (2017) designed a novel biological functional protein based on the *listeria* innocua Dps protein (named TDG1). This artificial caged protein containing gold-binding peptides can bind to gold nanowire and selectively deposit a thin layer of TiO_2_ on the gold surface. TiO_2_-coated gold nanowire can enable a more efficient collection of photogenerated electrons in perovskites and lower electrode resistance, thereby enhancing the performance of PSCs. Das developed a novel bio-PSCs structure to enhance the performance of PSCs by protein functionalization of TiO_2_. They immobilized bacteriorhodopsin (bR) protein molecules on the TiO_2_ surface, which performed the steps of carrier transport and reduced interfacial charge recombination. Through the forster resonance energy transfer (FRET) process, photogenerated electrons can be transferred from the perovskite layer to the bR molecules more efficiently. The experimental results showed that the PSCs with TiO_2_/bR structure achieve a PCE of 17.02% (2.41% higher than the reference device without bR structure), a J_SC_ of 22.61 mA cm^−2^, and a V_OC_ of 1.02 V. Due to the similar optical gap between bR molecules and perovskites, the electronic recombination loss is reduced, the carrier lifetime is increased, and thus the performance of PSCs is improved, which is conducive to the development of high-performance, high-stability, low-cost, and environment-friendly new biological perovskite solar cells (Das et al., 2019).

The application of natural protein in biological resources in the photovoltaic system has the advantages of rich resources, non-toxic, and sustainability. However, the lack of charge-transferring capacity and poor stability of natural proteins in cells hinder their development in the field of photovoltaics. Therefore, some artificial proteins have been more widely used, which can help ETL materials in PSCs transfer charge more efficiently. In addition, the barrier layer composed of protein can block the recombination of holes and electrons and improve the efficiency of the device.

### DNA

DNA (deoxyribonucleic acid) carries the genetic information necessary for the synthesis of RNA and proteins, and is an essential biological macromolecule for the development and normal functioning of organisms. In the molecular structure of DNA, two strands of deoxynucleotides are coiled around a common central axis, forming a double helix. Each nucleotide has a phosphate group, a pentose sugar, and a nitrogenous base. DNA has excellent thermal stability and favorable energy level (HOMO: −5.2 eV; LUMO: −1.1 eV), so it can be used in photovoltaic devices.

**FIGURE 9:**
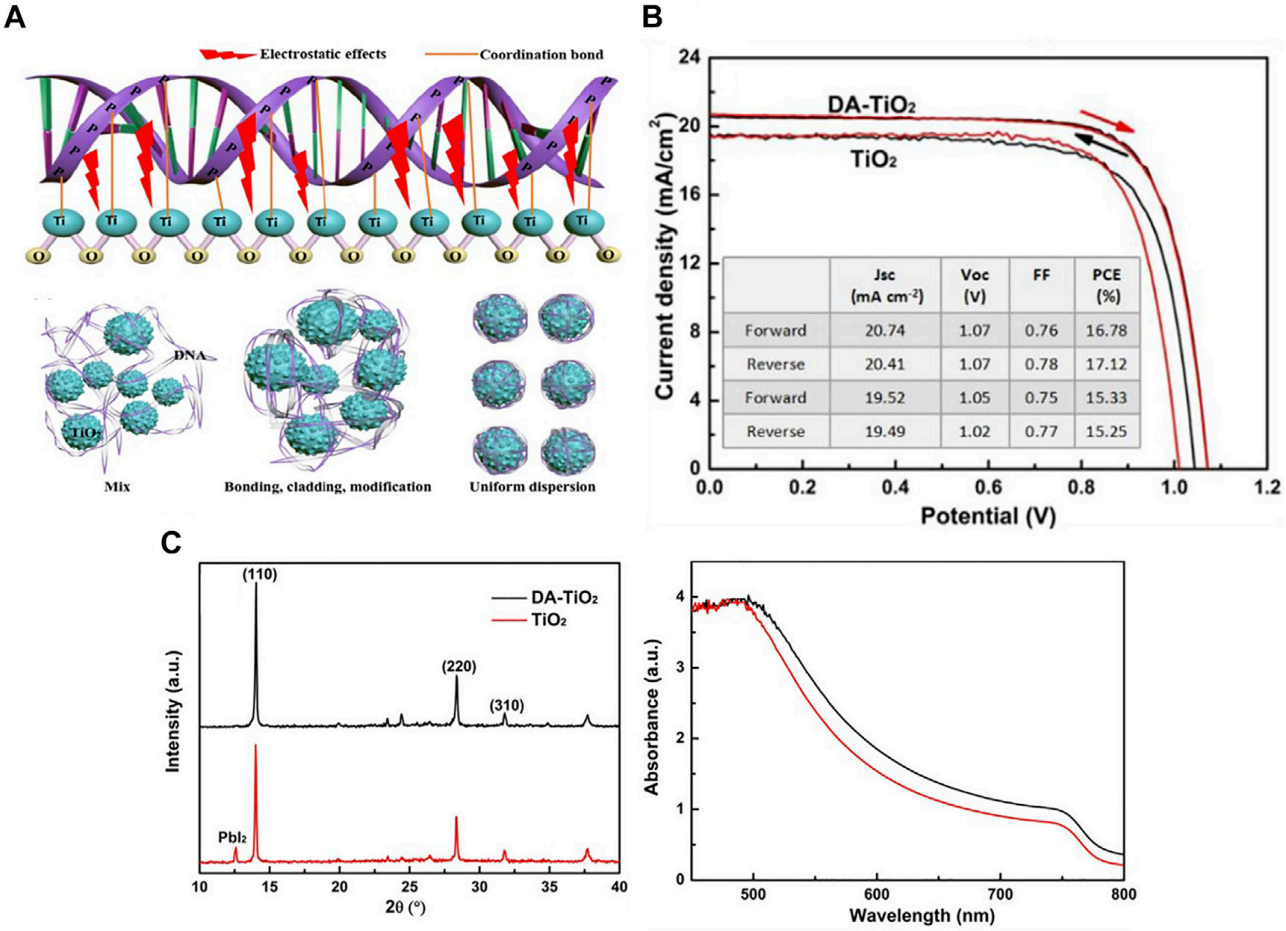
**(A)** The model diagram of the interaction between DNA and meso-TiO_2_, and the schematic diagram of the binding process of DNA and meso-TiO_2_. **(B)** Best efficiency of PSCs by reverse and forward scan directions. **(C)** XRD patterns and UV-vis absorption spectra of perovskite films deposited on dopamine (DA)-TiO_2_ and TiO_2_ ETL.

Yusoff fabricated PSCs at low temperatures using DNA-hexadecyltrimethylammonium chloride (CTMA) molecules as hole transport and electron blocking layers. The photoelectric conversion efficiency value of the perovskite solar cell using the bio-based charge transport layer was 15.86%, and the device still maintained 85% of the initial efficiency after 50 days in the air. DNA-CTMA perovskite solar cells can reduce the current hysteresis and significantly improve the lifetime in the air environment (Yusoff et al., 2016). Peng added different concentrations of DNA as a dopant into mesoporous TiO_2_ (meso-TiO_2_), and prepared DNA-doped mesoporous TiO_2_ as ETL by hydrothermal method. Figure 9A shows the interaction of DNA and meso-TiO2 and how they bind. It was found that the addition of DNA greatly improves the surface defects of mesoporous TiO_2_ and significantly reduces the carrier recombination rate. Several groups of DNA experiments with different concentrations showed that when the DNA concentration was 0.2 mg/ml, the photoelectric conversion efficiency of PSCs reached the highest (17.59%), and the device had higher short-circuited current density, open-circuit voltage, and filling factor. On the other hand, doping was conducive to the energy level matching between layers and could increase the charge transport efficiency in the device. The meso-TiO_2_ was obtained by hydrothermal reaction and did not need to be annealed at 500°C like TiO_2_ ETL (Peng et al., 2020).

Compared with other biological materials, DNA has favorable energy levels, which enables it to help ETL materials transport charges efficiently. At the same time, DNA has excellent thermal stability, so it has good compatibility in the preparation process of the device. DNA-doped TiO_2_ as ETL not only improves the performance of PSCs, but also does not complicate the fabrication process of the device, which reflects the potential of DNA in photovoltaic devices and can facilitate the commercialization of PSCs.

### Dopamine

Dopamine is a neurotransmitter, a chemical used to help cells transmit pulses. Dopamine is mainly used in the interface modification layer to improve electron transport efficiency and reduce charge recombination. Xue et al. (2018) achieved high-performance and high-stability planar perovskite solar cells through a dopamine semiquinone radical-modified layer. At the same time, hydroxyl groups can make the structure of perovskite crystal more stable. Hou introduced dopamine as a modification layer between SnO_2_ ETL and perovskite layer. This dopamine has two functional hydroxyl groups and amino groups. The amino groups can promote the growth of perovskite crystals and improve the surface defects of perovskite. The electron transfer of the dopamine-modified PSCs was significantly accelerated. The PCE of the device reached 16.87%, and the stability of the device was stronger (Hou et al., 2018). Zhai et al. (2018) demonstrated an *in-situ* interface engineering. In the process of synthesizing TiO_2_ nanoparticles, dopamine molecules were fixed on the surface of TiO_2_ and used as the ETL of planar PSCs. The presence of dopamine can optimize the band arrangement, inhibit the charge accumulation at the interface, and absorb the residual water on the surface. As can be seen from Figure 9B, the dopamine-treated device performed better. The experimental device structure was FTO/dopamine-TiO_2_/perovskite/spiro-OMeTAD/Ag, and finally the PCE of this device achieved 16.36%. Figure 9C shows that when the perovskite material was deposited on the dopamine-modified substrate, the perovskite diffraction peak was significantly enhanced, indicating that the crystallinity of the perovskite film had been significantly improved, and at the same time, stronger UV-vis absorption was observed on DA-TiO_2_ device. In 2019, Liu et al. (2020) prepared high-quality perovskite crystals through dopamine modification, optimized the functional conversion efficiency of planar PSCs to 19.45%, and confirmed that the dopamine *in situ* functionalization of TiO_2_ can optimize the energy level arrangement and enhance the charge extraction, thus reducing the charge accumulation at the interface. Zhang Y. et al. (2019) took the TiO_2_ nanoparticles modified by dopamine as the electron transport layer of PSCs. The dopamine-modified TiO_2_ improved the binding with perovskite active layer through chelation. In the process of solution deposition of PSCs, dopamine can reduce the oxygen vacancy in TiO_2_, and the amino group can also passivate the uncoordinated lead atoms in halide perovskite and improve the interface defects. The champion cell exhibited a PCE of 20.93%. In terms of stability, after 1,200 h of operation under sunlight (nitrogen environment), the battery efficiency could still maintain 80%, which is an important part of the commercialization of PSCs in the future.

In the ETL of TiO_2_ nanoparticles, the addition of dopamine can facilitate the direct injection of photogenerated electrons into the conduction band of TiO_2_, thereby reducing the charge accumulation at the interface. In addition, dopamine can tune the energy level arrangement of TiO_2_ and enhance charge extraction (Zhang and Yu, 2021). Except for the mesoporous structure, the use of dopamine-functionalized interfaces in planar PSCs can improve the charge extraction ability of metal oxide ETL and enhance the quality of perovskite films.

We listed several typical biomaterials that can be used for ETL preparation. In addition to these materials, some other materials also have good application prospects. For example, catecholamine is an ideal material for ETL because of its strong adhesion capability and chemical stability. Catecholamine is rich in functional groups and can effectively functionalize the surface of the film. It has been successfully used in supercapacitor and drug delivery of biological therapy (Cavinato et al., 2021). Moreover, carotenoids, as natural organic pigments, are ideal substitutes for p-type semiconductors. Carotenoids have an efficient light harvesting mechanism and strong charge transfer capability, making them possible for their development in solar cells. However, the instability of natural pigments is a problem that cannot be ignored.

In conclusion, due to the abundant functional groups in bio-derived materials, they can help common ETL materials to efficiently collect and transport charges, and PSCs devices modified with these materials achieve better performance (Cavinato et al., 2021). Moreover, in addition to the performance enhancement, these modifications can also promote the improvement of device stability, which is crucial for the commercial production of PSCs. Despite the success of bio-based PSCs, in addition to the above materials, there are still many other bio-derived materials to be explored. Regardless of the device structure, bio-derived materials can serve as an option to enhance the performance of PSCs. It is worth noting that these materials are all introduced into PSCs as additives, which play auxiliary functions and do not change the bulk properties of the device.

## The Application of ETL in Bioengineering

Chemistry and biology are two closely related disciplines. The application of bio-derived materials in PSCs has well reflected this point. Similarly, PSCs materials also have many applications in bioengineering. Up to now, most studies focus on the biological application of perovskite materials, which is due to the excellent properties of perovskite single crystal. Moreover, because perovskite materials in PSCs have adjustable energy band structures, the crystal structure can be changed by molecular design to meet the needs of various applications. We illustrate the application of perovskite materials in PSCs for bioengineering through biological detection, biological imaging, and biomedical treatment. Among them, bio-detection and bio-imaging can provide key information for medical research or disease diagnosis, and biomedical treatment is mainly aimed at new treatment methods based on nanotechnology.

### Biological Detection

Photoelectric properties of perovskite crystals make them ideal for biological detectors. Biological detection mainly monitors chemical components in biological samples employing biosensors, X-rays, etc. Bio-sensors have the functions of receivers and converters, in which PSCs can act as transducers, converting optical signals into electrical signals. And the halide perovskite crystals with unique luminescent properties are attractive for fluorescent biology. Due to the existence of heavy atoms in perovskite materials, they have shorter X-ray absorption lengths and better detection efficiency. Therefore, perovskite materials have a good application prospect in the application of X-rays.

Wei et al. (2016) found that X-ray detectors made from perovskite single crystals showed ultrahigh sensitivity, four times higher than those of commercial α-Se X-ray detectors. This has confirmed the application prospect of perovskite materials in X-ray detection. Although perovskite crystals perform well in X-ray detectors, the integration of perovskite single crystals and 2D pixels on commercial chips is required for biomedical detection technology applications (Sun et al., 2020). Therefore, the road to the commercialization of this research is still in its infancy. Compared with perovskite crystals, perovskite quantum dots (QDs) also have applications in bioengineers. Functionalized QDs can be used to observe and calculate the concentration and distribution of biomarkers in organisms. In addition, QDs can be used as cell sensors to detect cancer cells through fluorescence and electrochemical signals (Cheng et al., 2021).

The application of perovskite materials in biomonitoring reflects the close connection between PSCs and bioengineering. The ETL metal oxide material also has a good performance in biological monitoring. Metal oxides are versatile enough to monitor molecules from biological systems. Taking biosensors as an example, we will enumerate the applications of TiO_2_, SnO_2,_ and ZnO in bioengineering. TiO_2_ is usually used in biomonitoring in the form of mesoporous films. Mesoporous TiO_2_ exhibits excellent biosensing properties, including high sensitivity, broad linear response, and good reproducibility. Therefore, mesoporous TiO_2_ biosensors have promising applications in glucose detection, hydrogen peroxide detection, cholesterol detection, and pancreatic cancer detection (Amri et al., 2021). SnO_2_ is an n-type semiconductor with a high specific surface area, good chemical stability, and biocompatibility. And SnO_2_ nanoparticles have applications in many biosensing fields. The SnO_2_ nanoparticles prepared by the precipitation method can be used for the detection of l-cysteine. By using sonication method, the SnO_2_ nanoparticles can be employed for pesticide detection. To be used for hydrogen peroxide detection, SnO_2_ nanoparticles need to be prepared by microwave irradiation (Serban and Enesca, 2020). ZnO has piezoelectric properties based on a non-centrosymmetric crystal structure, and compared with SnO_2_, ZnO has better binding ability with living organisms. In addition, ZnO’s non-toxicity and compatibility with human skin enable it to be a permanent human sensor for disease treatment. Mei reported the utilization of ZnO thin films and nanostructures to enhance the performance of surface plasmon resonance sensors for the detection of biological and chemical compounds. In the future, ZnO nanostructures have promising potential applications in highly sensitive miniature biosensors (Mei et al., 2020). ETL metal materials are considered to be multifunctional materials that can be successfully integrated into biosensor technology. In addition, the high stability and biocompatibility of these materials make them promising for application in smart biosensing devices.

### Biological Imaging

Medical imaging enables medical diagnosis by revealing organ abnormalities or tumor lesions. To obtain high-contrast images, the application of contrast agents is crucial. Contrast agents have special optical and magnetic properties, and they can produce signal enhancement for specific imaging results. In various medical imaging techniques, a variety of contrast agents have been developed. At the same time, materials in PSCs have also been developed as contrast agents.

**FIGURE 10:**
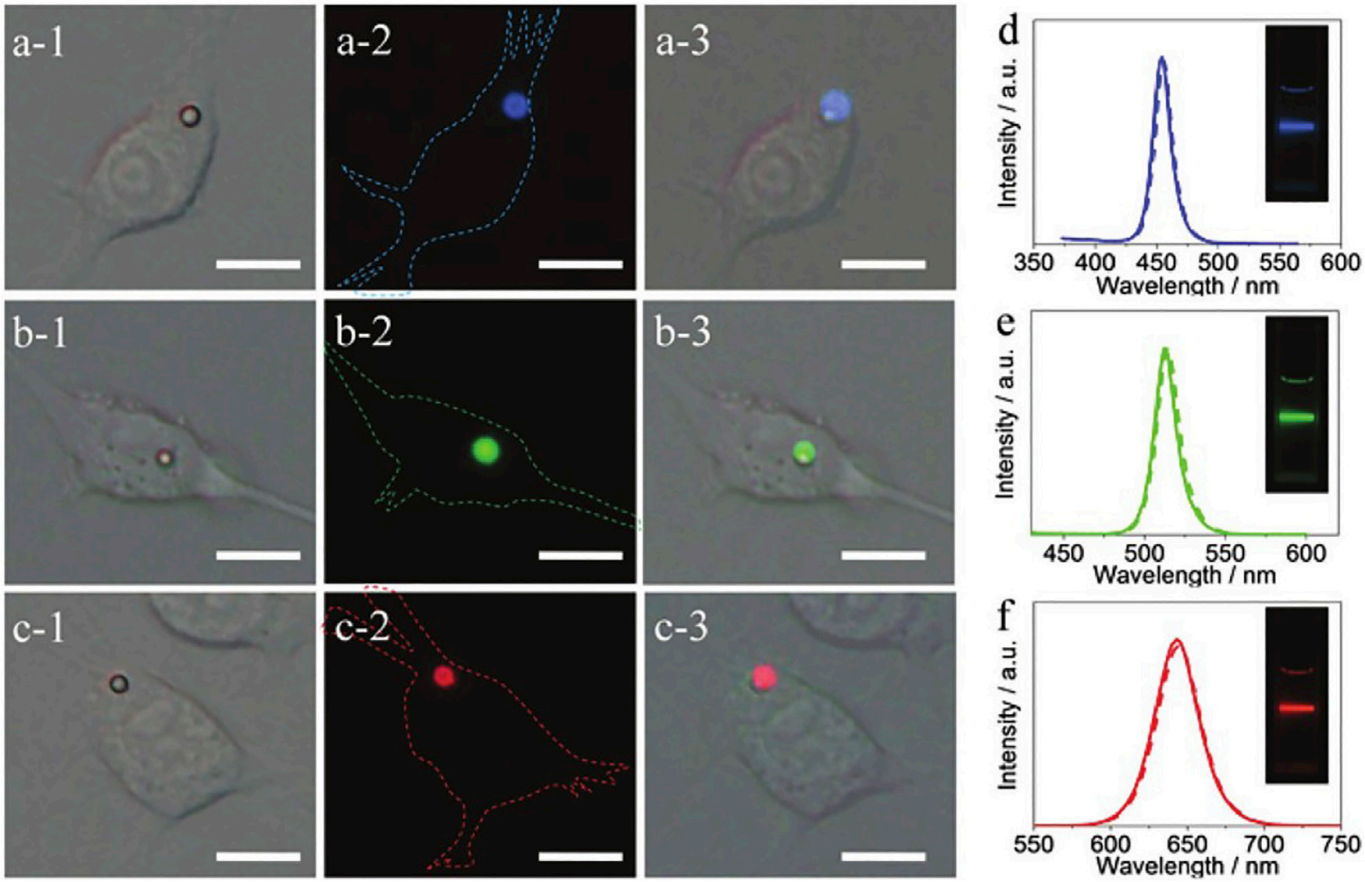
**(A,B,C-1)** and **(A,B,C-2)** are bright-field and fluorescence images of RAW 264.7 cells incubated with CsPbX_3_ NCs@MHSs. **(A,B,C-3)** are superimposed pictures of (-1) (-2). **(D–F)** show the fluorescence spectra of CsPbX_3_ NCs@MHSs (dotted line) and the corresponding NCs colloidal solution (solid line).

Halide perovskite crystals are ideal as contrast agents for biological imaging due to their bright luminescence and broad spectral tunability. Zhang et al. (2017) prepared highly luminescent colloidal CsPbX_3_ (X = Cl, Br, I) using a solution self-encapsulation method at room temperature and used PVP as the encapsulation ligand to reduce the contact of the crystal with moisture in the air. They embedded CsPbX_3_ nanocrystals (NCs) into the microsphere (MHSs) of polystyrene matrix to prepare CsPbX_3_ NCs@MHSs mixture as multicolor multiplexing optical coding agent. They cultured macrophages with medium and phosphate saline containing CsPbX_3_ NCs@MHSs. After 24 h of culture, confocal fluorescence images observed that NCs@MHSs entered living cells with strong intracellular emission under the excitation of 325–375 nm radiation. Experiments demonstrate that NCs@MHSs can be used as tunable luminescent probes for bioanalysis (Figure 10). Of course, the research only shows the application prospects of perovskite crystal materials in bioluminescence and bioimaging, the toxicity of lead elements in perovskite, and instability in water hinder its development in this field.

Metal oxide materials used in ETL can be used as materials for metal-based contrast agents in biological imaging. These applications are mainly about metal oxide nanomaterials because of the advantages of nanomaterials in tunability and photostability. Among the several materials introduced in Chapter 3, TiO_2_ nanoparticles are the most widely used in bioimaging due to their biological sensitivity, strong binding ability, and little effect on living cells. TiO_2_ has been demonstrated to be a photocatalyst and can strongly absorb near-infrared light waves (Anderson et al., 2019). To enhance the imaging capabilities of TiO_2_, some studies combine it with other molecules as contrast agents (Wang et al., 2018). The tunability of these materials in size, structure, and chemical properties makes them more promising in bioimaging. Currently, how to control and improve the performance of metal oxide nanoparticles to meet the requirements of bioimaging is the focus of research.

### Biomedical Treatment

With the progress of molecular medicine and nanotechnology, treatment *in vivo* is no longer limited to traditional drug treatment. New treatment methods can use bio-compatible nano-devices implanted into the human body to realize personalized treatment. These medical nano-devices need electric energy to perform their functions in the human body. The energy acquisition mode of the devices may be the kinetic energy provided by blood flow, the chemical energy released by biological tissues, and the light energy obtained by nano-photovoltaic devices. Because there is a near-infrared window from 650 to 1350 nm (called the “treatment window”), in which photons can enter human tissue, the absorption of medical photovoltaic equipment can be changed through molecular design to adapt to the area above, to obtain photons that can penetrate the tissue (Chen, 2019). However, the traditional photovoltaic devices do not have bio-compatibility and cannot be directly implanted into the human body. Therefore, the device materials need to be changed by molecular modification. Moreover, photovoltaic cells cannot effectively absorb photons in the treatment window, which is the reason that hinders their developments in biomedicine. Based on this, Chen et al. (2009) reported 980 nm laser-driven photovoltaic cells based on rare-earth up-converting phosphors. Rare-earth phosphors, such as some transition metal compounds, can help photovoltaic cells effectively absorb near-infrared photons. The penetration depth of 980 nm photons in biological tissues is up to several centimeters, which makes the application of photovoltaic cells in treatment *in vivo* possible. Although the PCE value of PSCs is excellent in photovoltaic devices, their instability is difficult to solve. For example, perovskite crystals are very sensitive to moisture/oxygen in the air. In addition, due to the toxicity of perovskite materials, it has many limitations in some biological applications. One solution is to use other non-toxic cations to replace lead and reduce the damage of lead. For the application of ETL in biomedicine, the relevant research is still in a relatively early stage. To achieve specific biomedical functions, it is necessary to not only change the molecular structure of materials, but also improve the light absorption efficiency of light absorbers in a specific spectral range.

In recent years, metal oxide nanoparticles have achieved good application results in biomedical treatment, and they have developed rapidly as biomedical materials in immunotherapy, tissue therapy, and regenerative medicine. The application of metal nanoparticles to medical treatment embodies many advantages of modern medicine. Of course, ETL metal nanomaterials are also important biomedical materials. Due to its ability to induce cell adhesion, TiO_2_ has been used as a biomedical material for wound healing and bone integration (Haugen et al., 2013). In terms of drug therapy, TiO_2_ is often used to deliver drugs due to its simple active release mechanism for the purpose of binding to target molecules. Moreover, the biodegradable properties of ZnO nanoparticles and their cytotoxicity to cancer cells are commonly used to deliver cancer drugs. In addition, since zinc is involved in insulin synthesis, secretion, and storage, ZnO nanoparticles have the potential to be a drug for alleviating diabetic complications (Nikolova and Chavali, 2020). Drug delivery with nanocarriers has advantages in efficiency, accuracy, and stability. To reduce the toxicity of nanomaterials and alleviate side effects for patients, a combination of engineered materials and nanomaterials is required. As a result, scientists are often faced with a balance between high medical properties and toxic side effects. To develop more efficient and precise therapeutic devices, it is crucial to develop new metal nanomaterials with low toxicity and high biomedical properties.

## Conclusion

In recent years, perovskite solar cells have made rapid progress in terms of efficiency, performance, and stability. With the development of PSCs, in-depth research on various aspects of ETL is crucial. ETL improves the transmission efficiency of electrons and provides a guarantee for the quality and stability of the device. In this review, we provide an overview of the structures and materials of PSCs, and summarize the relationship between PSCs and bioengineering. At present, ETL materials have some problems, such as energy level mismatch, surface defects, and insufficient fluidity. Scientists have tried element doping, surface modification and other ways to change material properties. In response to the problems of existing materials, the development of new materials for ETL is another direction of future research. At the same time, biomaterials have played a great role in the preparation of ETL, and they can assist traditional materials for efficient charge transport. The new bio-PSCs structure uses natural biomaterials for light energy conversion, which provides an excellent idea for the commercialization of environment-friendly photovoltaic devices.

Future solar cells may develop in the direction of flexible, transparent and lead-free perovskite devices. Some current materials can no longer meet the demand. Continuing to explore new materials and change the device structure may be the direction of future research. In future research, the commercialization of bio-PSCs can be accelerated by improving various aspects of ETL, making progress in improving charge transfer efficiency and reducing charge collection loss, and developing bio-PSCs with practical application value. Realizing the close connection between the ETL of PSCs and bioengineering requires researchers to overcome many difficulties. We look forward to more researchers from the fields of chemistry, biology, and medicine, paying attention to the problems in the combination of PSCs and bioengineering at this stage, and promoting the practical application of perovskite materials in bioengineering.
